# Supplementary figures and images for: Automatic detection, identification and counting of anguilliform fish using in situ acoustic camera data: Development of a cross-camera morphological analysis approach
Source: PLoS One. 2023 Feb 24;18(2):e0273588. doi: 10.1371/journal.pone.0273588 (PMC9956004; doi:10.1371/journal.pone.0273588)

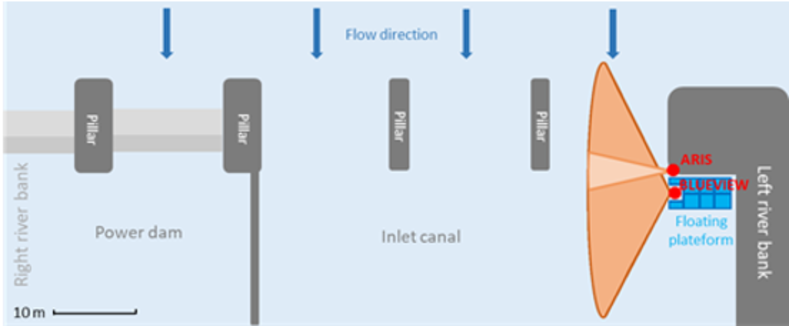

Supplement: S1 Fig — (TIF) [file pone.0273588.s001.tif]

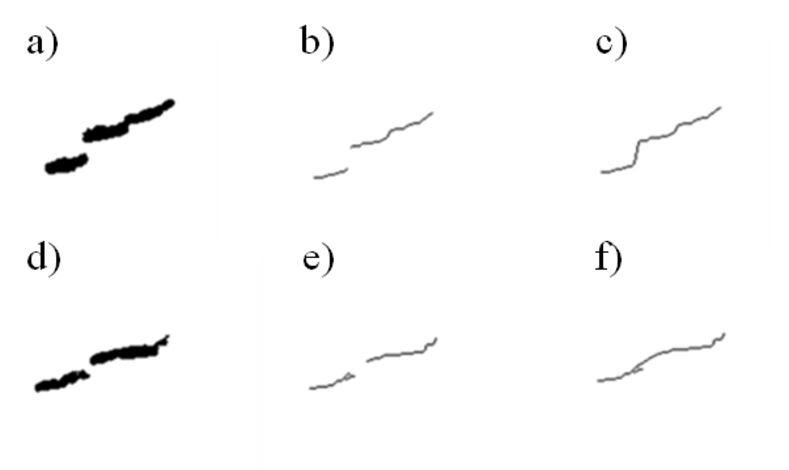

Supplement: S2 Fig — a) Binary image of the candidate’s body after background subtraction; b) Binary image of the candidate skeleton; c) Binary image of the candidate reconstructed skeleton. (TIF) [file pone.0273588.s002.tif]

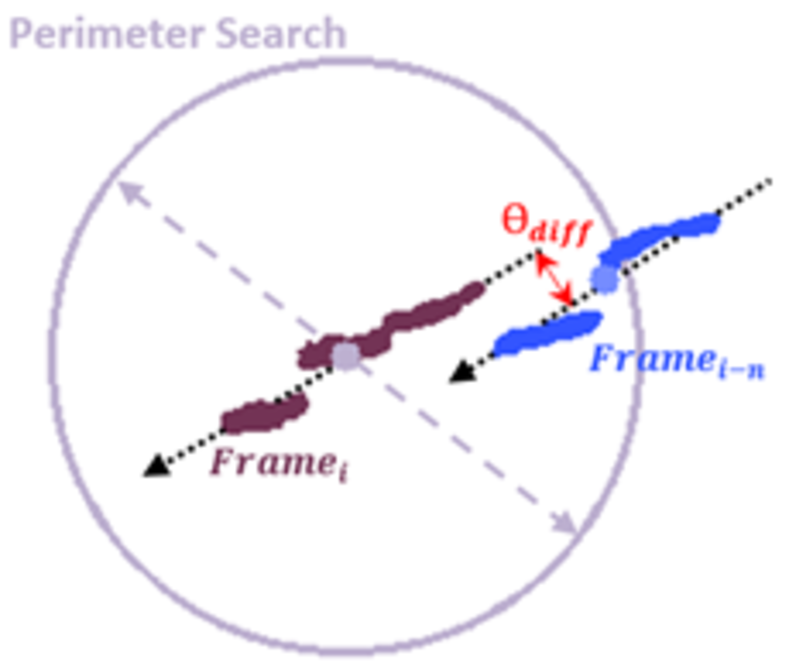

Supplement: S3 Fig — (TIF) [file pone.0273588.s003.tif]

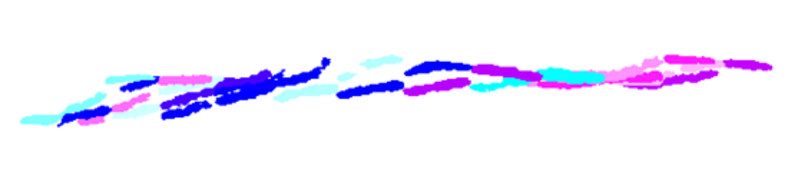

Supplement: S4 Fig — (TIF) [file pone.0273588.s004.tif]

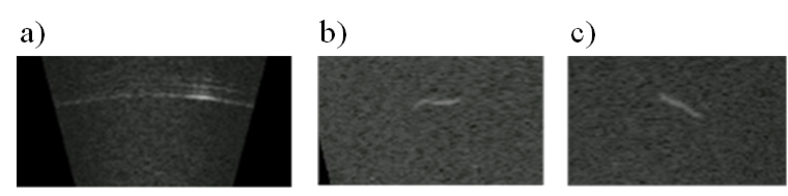

Supplement: S5 Fig — a) Example of an arcing effect; b) Example of the method misidentification due to fish swimming closely to each other, c) Example of the method misidentification as an eel of another fish species. (TIF) [file pone.0273588.s005.tif]
