# Supplementary material for: Automatic detection, identification and counting of anguilliform fish using in situ acoustic camera data: Development of a cross-camera morphological analysis approach
Source: PLoS One. 2023 Feb 24;18(2):e0273588. doi: 10.1371/journal.pone.0273588 (PMC9956004; doi:10.1371/journal.pone.0273588)
Supplement: S1 File — (PDF) [file pone.0273588.s006.pdf]

S1 File. Calculation of echograms of singular values ratios

The different steps of the echogram generation (Fig 2) are described below.

Frames are processed one after the other. A pre-treatment is firstly carried out on each of them. To minimize the impact of noise in the analysis as well as to highlight the objects which could merge with the ambient background, due to their orientation towards the camera, a Lee filter (S1) is applied on each analysed frame. It will perform smoothing by minimizing either the mean square error or the weighted least square estimation. Smoothing is performed only if the area variance is low, preserving low and high contrast (1).

(S1) $Y_{ij} = \overline{K} + W * (C_{ij} - \overline{K})$

Where, $\overline{K}$, the kernel mean ; C_{ij}, the value of the calculated pixel ; W, the weight function : $W = \frac{\sigma_K{}^2}{\sigma_K{}^2 + \sigma^2}$; $\sigma_K{}^2$, the kernel variance ; σ^2, the image variance.

The frame is then analyzed by performing a succession of singular value decomposition. Square sliding windows are scanning the frame with 50% overlap. Window size, d_w, is defined according to the frame resolution r and to the minimum length targeted L_{eel_min} (S2). Their number, W_1 along the horizontal axis and W_2 along the vertical one, thus depend on the frame dimension D_1 x D_2 (S3). A singular value decomposition is therefore systematically carried out on the successive part of the image covered by the sliding windows. At each slide of the window, ratio of each analyzed singular value is extracted according to a reference image (S4), a standard image obtained from the same acoustic camera with no objects passing through, thus without any discontinuities. This information is recorded in a matrix of dimension W_1 x W_2. To reduce the impact of the camera's edge effects, a mask is calculated relatively to the reference image (S5). It is then applied (S6) on each singular value ratio matrix.

(S2) $d_w = \frac{L_{eels\_min}}{r}$

(S3) $W_1 = \frac{2*(D_1 - d_w)}{d_w}$, $W_2 = \frac{2*(D_2 - d_w)}{d_w}$

(S4) $\forall$ w $\in [1, W_1], \forall$ v $\in [1, W_2],\ S_{w,v} = \frac{\sigma_N^{w,v}}{\sum_{n=1}^{n=N} \sigma_n^{w,v}} - \frac{\sigma_{N,ref}^{w,v}}{\sum_{n=1}^{n=N} \sigma_{n,ref}^{w,v}}$

(S5) $\forall$ w $\in [1, W_1], \forall$ v $\in [1, W_2],\quad M_{w,v} = \min\left(I_{i,j,ref}\right)_{\substack{(w-1)*\frac{d_w}{2} \leq i \leq (w-1)*\left(\frac{d_w}{2}\right)+d_w \\ (v-1)*\frac{d_w}{2} \leq j \leq (v-1)*\left(\frac{d_w}{2}\right)+d_w}}$

(S6) $\forall$ w $\in [1, W_1], \forall$ v $\in [1, W_2],\ R_{w,v} = S_{w,v} * M_{w,v}$

Where L_{eel_min}, the minimum targeted length; r, the resolution of one pixel in millimetres; D_1 and D_2 the dimension of the frame in pixels; d_w, the square sliding window side dimension in pixels; W_1 and W_2 the number of window along the vertical and horizontal axes; $\sigma_N^{w,v}$, the Nth singular value of the window (w, v); $\sigma_{N,ref}^{w,v}$, the Nth singular value of the window (w, v) of reference; S, the ratio matrix; M, the mask ; R, the ratio result matrix; I_{ref}, the reference frame

From these results matrix is kept the maximum of the discontinuities ratios (S7) along the vertical axis, i.e. along the main axis of the camera FOV. This allows us to study the discontinuities present over the entire depth of the camera, perpendicularly to the axis of the flow. These maximum values progressively build up the intermediate echogram for each singular value studied. One column thus represents one frame of the video. Echogram’s dimension matches the number of frames of the video by the number W_2 of sliding windows along the vertical axis of the frames.

(S7) $\forall$ i $\in [\![1, W_2]\!],\ V_{i,1} = \max\,(R_{i,j})_{1 \leq j \leq W_1}$

Where V, the matrix of maximum ratios along the vertical axis.

The two resulting echograms are merged, using an adaptive threshold, function of the average value of the ratios at each range. It allows the extraction of objects detected simultaneously and the deduction of the frame intervals to be further examined.

References

1. Lee J. Digital Image Enhancement and Noise Filtering by Use of Local Statistics. IEEE Transactions on Pattern Analysis and Machine Intelligence. 1980;PAMI-2(2):165–8.
