# Supplementary material for: Automatic detection, identification and counting of anguilliform fish using in situ acoustic camera data: Development of a cross-camera morphological analysis approach
Source: PLoS One. 2023 Feb 24;18(2):e0273588. doi: 10.1371/journal.pone.0273588 (PMC9956004; doi:10.1371/journal.pone.0273588)
Supplement: S2 File — (PDF) [file pone.0273588.s007.pdf]

S2 File. Reconstruction and extraction of skeleton characteristics

The skeleton calculation is performed by successively removing the pixels located on the borders as long as this does not break the connectivity of the object studied (1), preserving the shape with a minimum number of pixels. To perform this transformation, candidates are split into two categories depending on the number of items that compose them. The simplest case is where the candidate has been recorded by the camera in a single entity. Skeleton is calculated and no further process is necessary. In the other case, the object is potentially captured in several entities (S1a and S1d Figs). This happens frequently when it comes to eels due to the ripple and the orientation of their body relative to the camera beam resulting in fragmented images. It is thus necessary to reconstruct the skeleton (S1b and S1e Figs) in order to recover more reliable and closer to reality physical characteristics. Extreme points along the main axis of each item are calculated. They are then linked with those of the other closest item respecting a pre-define distance and degree of difference. Once the skeleton is fully retrieved (S1c and S1f Figs), it is possible to extract its characteristics.

References

1. Lü H, Wang P. A comment on a fast parallel algorithm for thinning digital patterns. Communications of the ACM. 1986;29:239–42.
